# Phase II trial of modified FOLFOX6 and erlotinib in patients with metastatic or advanced adenocarcinoma of the oesophagus and gastro-oesophageal junction

**DOI:** 10.1038/bjc.2011.280

**Published:** 2011-08-02

**Authors:** Z A Wainberg, L-S Lin, B DiCarlo, K M Dao, R Patel, D J Park, H-J Wang, R Elashoff, N Ryba, J R Hecht

**Affiliations:** 1 Division of Hematology/Oncology, Department of Medicine, Geffen School of Medicine at UCLA, Los Angeles, CA, USA; 2 Translational Oncology Research International (TORI), Los Angeles, CA, USA; 3 Department of Biostatistics, Geffen School of Medicine at UCLA, Los Angeles, CA, USA

**Keywords:** oesophagogastric cancer, erlotinib, EGFR, FOLFOX

## Abstract

**Background:**

There is increased recognition that cancers of the upper GI tract comprise distinct epidemiological and molecular entities. Erlotinib has shown activity in patients with adenocarcinoma of the oesophagus/gastro-oesophageal junction (GEJ), but not in distal gastric cancer. mFOLFOX6 is one of several active regimens used to treat adenocarcinoma of the Eso/GEJ. This study evaluates the efficacy and safety of mFOLFOX6 and erlotinib in patients with metastatic or advanced Eso/GEJ cancers.

**Methods:**

Patients with previously untreated advanced or metastatic Eso/GEJ adenocarcinoma are treated with oxaliplatin 85 mg m^–2^, 5-FU 400 mg m^–2^, LV 400 mg m^–2^ on day 1, 5-FU 2400 mg m^–2^ over 48 h and erlotinib 150 mg PO daily. Treatment was repeated every 14 days. The primary objective was response rate (RR), secondary objectives include toxicity, progression-free survival (PFS), overall survival (OS) and to correlate clinical outcome with expression patterns and molecular alterations in the epidermal growth factor receptor-dependent pathways.

**Results:**

A total of 33 patients were treated and evaluable: there were two complete responses, 15 partial responses for an objective RR of 51.5% (95% CI, 34.5–68.6%). Median PFS was 5.5 months (95% CI, 3.1–7.5 months) and median OS was 11.0 months (95% CI, 8.0–17.4 months). The most common grade 3–4 toxicities were: diarrhoea (24%), nausea/vomiting (11%), skin rash (8%) and peripheral neuropathy (8%). The frequency of alterations was KRAS mutations (8%), EGFR mutations (0%) and HER2 amplification (19%).

**Conclusion:**

In patients with Eso/GEJ adenocarcinoma, mFOLFOX6 and erlotinib is active, has an acceptable toxicity profile and FOLFOX±erlotinib could be considered for further development.

Adenocarcinoma of the stomach is the most common gastrointestinal cancer in the world and the second leading cause of cancer death worldwide (Jemal *et al*, 2009). Although there is significant geographic variation in this disease, recent trends in incidence have suggested that gastro-oesophageal junction (GEJ) adenocarcinomas are among the fastest growing malignancies in the Western world (Blot and McLaughlin, 1999). Furthermore, despite significant evidence that adenocarcinomas of the GEJ have distinct epidemiologic and pathologic features; they are often grouped with distal gastric cancers in clinical studies (Marsman *et al*, 2005). Therefore, novel investigational approaches are needed for this subset of upper gastrointestinal tract cancers in order to improve outcomes.

In patients with metastatic gastric and oesophageal adenocarcinomas, the median overall survival (OS) ranges between 7 and 10 months. Most studies in oesophageal and gastric cancers have included fluoropyrimidines and platinums as the backbone of these therapies, with some regimens containing a third chemotherapeutic agent. A randomised phase III trial demonstrated that the combination of fluorouracil and oxaliplatin was at least equivalent to if not better than fluorouracil and cisplatin with an improved toxicity profile (Al-Batran *et al*, 2008). In addition, the REAL-2 trial demonstrated that the triplet regimens, which contained oxaliplatin in place of cisplatin showed similar efficacy. In these trials, the median OS times were 10.7 and 11.2 months, respectively (Cunningham *et al*, 2008).

The epidermal growth factor receptor (EGFR) is a transmembrane glycoprotein that is part of the human EGFR (HER) family. As in many epithelial malignancies, oesophageal and gastric cancer studies have shown significant variability in overexpression of EGFR with rates ranging between 17% and 90% (Takehana *et al*, 2003; Hanawa *et al*, 2006; Pinto *et al*, 2007). Many of these studies have shown that overexpression of the EGFR is correlated with a worse prognosis (Ozawa *et al*, 1989; Wang *et al*, 2007). Therefore, it has been speculated that EGFR blockade may be an effective therapeutic strategy in this disease.

This phase II trial was designed to expand on two previous trials that investigated the role of EGFR tyrosine kinase inhibitors in upper GI adenocarcinomas (Dragovich *et al*, 2006; Ferry *et al*, 2007). Both these single-agent trials demonstrated that there was modest activity in patients with tumours that were derived from the oesophagus and GEJ with no objective responses seen in patients with distal stomach cancer. The current trial was designed to determine the efficacy and toxicity of FOLFOX and erlotinib specifically in patients with oesophagus and GEJ adenocarcinoma with a particular emphasis on possible surrogate biomarkers.

## Patients and methods

### Eligibility

Patients with a histologic diagnosis of adenocarcinoma of the oesophagus or GEJ with measurable disease by Response Evaluation Criteria in Solid Tumours (RECIST) were considered eligible for the trial. In this study, oesophagus and GEJ tumours were defined by the Siewert classification (Siewert class I, II and III), which included cancers arising within 5 cm of the anatomic GE junction (distal oesophagus) or from the gastric cardia (Siewert and Stein, 1998). All patients enrolled on this study had an assessment of the GEJ made by imaging and by review of an endoscopic report. Patients with gastric cancers that did not involve the GEJ were excluded from participation.

Only patients with previously untreated metastatic disease were included. Previous radiotherapy or adjuvant chemotherapy was permitted as long as no treatment was received in the previous 12 months. Other eligibility requirements included ECOG performance status of 0 or 1, peripheral neuropathy ⩽grade 1, the ability to swallow oral medications, adequate haematologic (absolute neutrophil count >1500 mm^–3^, platelets>100 000 mm^–3^), renal (creatinine clearance >60 ml min^–1^) and hepatic function (total bilirubin⩽1.5 × ULN, AST and ALT⩽3.0 × ULN or ⩽5.0 × ULN if there is liver metastasis). Patients with a history of CNS metastasis, a history of active infections or other concomitant serious medical conditions were excluded from participation. Approval from institutional review boards from each participating centre was obtained. This study has been completed and registered at ClinicalTrials.gov, number NCT00591123.

### Study design

This was a phase II open label, multi-centre trial administered under the not for profit TORI network. Erlotinib was provided by OSI Pharmaceuticals (Melville, NY, USA). The primary objective of the study was to assess overall response rate (ORR). Secondary objectives included assessment of toxicity, OS and progression-free survival (PFS), and exploratory analysis of translational endpoints in patient samples. Patients received modified FOLFOX6 (oxaliplatin 85 mg m^–2^, 5-FU 400 mg m^–2^, leucovorin 400 mg m^–2^, on day 1 followed by 5-FU 2400 mg m^–2^ over 48 h) and erlotinib 150 mg day^–1^ continuously. Two weeks constituted one cycle.

#### Treatment assessments

Baseline assessments included history, physical examination including neurological and skin exam, CBC and platelet count, serum chemistries and diagnostic tumour imaging. During the study, history, physical exam, performance status, CBC, chemistry and toxicity assessments were evaluated before the start of each cycle. Toxicity assessments were based on the National Cancer Institute Common Toxicity Criteria for Adverse Events, version 3.0 (http://ctep.cancer.gov/protoco lDevelopment/electronic_applications/ctc.htm). Tumour response measurements were done after every four cycles (8 weeks) and measurable lesions measured by CT scans were defined by modified RECIST criteria. Safety assessments consisted of regular monitoring of haematology, serum chemistries and physical exams with continuous recording of all adverse events (AE's).

#### Statistical design

The primary objective of this trial was to assess objective response rate (RR), defined as the proportion of patients with complete response (CR) or partial response (PR). The objective RR and its 95% CI were estimated. Based on an ORR of 34.8%, which was reported in the largest randomised trial that used the doublet of 5-FU and oxaliplatin, a target RR for the combination of FOLFOX and erlotinib was defined as >44% to viewed as promising (Al-Batran *et al*, 2008). A planned sample size of 38 eligible patients would then provide a 90% CI with a type 1 error of 10%. Progression-free survival was defined as the time from the date of initial treatment to first objective documentation of disease progression, or off-study, whichever came first, and OS was defined as the time from date of initial treatment to date of death or the last contact time if patient was still alive. Survival function was estimated using the Kaplan–Meier method and survival curves were generated for both PFS and OS. Data for all eligible and treated patients were done on all patients who received any treatment on this study.

#### Translational correlatives

Representative paraffin blocks were required for all participating patients. Tissue blocks were obtained on 36 patients. The objectives of the correlative studies were (1) to assess the status of mutations in EGFR, KRAS, BRAF and PI3 kinase, (2) to assess the rates of EGFR and HER2 amplification and HER3 expression and (3) to investigate whether any of these markers were predictors of clinical benefit (as determined by RR, PFS and OS). The association between biomarker and objective response was investigated using the Fisher's exact test.

The EGFR, KRAS, BRAF and PI3 kinase mutation testing was done by purifying DNA using the MagneSil Genomic Fixed tissue System DNA Isolation Kit (Promega, Madison, WI, USA). Tumour areas of interest were selectively microdissected from the section. DNA was isolated from the collected cells using an extraction protocol designed for small paraffin-embedded tissues. The DNA was added to individual allele-specific PCR reactions targeting seven mutations in codons 12 and 13 of KRAS, seven mutations in exons 9 and 20 of the PI3 kinase gene, the T>A transversion in exon 15 of the BRAF gene and the most prominent mutations, deletions and insertions in exons 18–21 of the EGFR gene. The KRAS mutation-specific reactions evaluate G12A, G12C, G12D, G12R, G12S, G12V and G13D whereas the PI3K panel covers E542K, E545K, E545G, E545D, E545A, H1047L and H1047R. The BRAF PCR targets the mutation associated with the V600E alteration and the EGFR panel includes reactions, which evaluate T790M, L858R, L861Q, G719A, G719S, G719C, S768I, 19 deletions in exon 19 and 3 insertions in exon 20. Each reaction is capable of detecting a mutation to 1–2% in a background of non-mutated DNA. All testing was performed using a real-time PCR platform (Clarient Inc., Alisa Viejo, CA, USA).

The EGFR and HER2 gene copy number were analysed using fluorescence *in situ* hybridisation (FISH) on 5 μm thick paraffin sections. HER-2 gene amplification was assessed using the commercially available PathVysion HER-2 DNA probe kit (Vysis Corp., Downers Grove, IL, USA), which utilises a locus-specific probe mapping to the HER-2 gene 17q11.2-q12 (orange) and one control probe (green) mapping to the centromere of chromosome 17. The probe for identifying EGFR gene amplification is a commercially available probe cocktail that maps to the EGFR gene 7p12 (orange), with a control probe that maps to the centromere of chromosome 7 (green) (Vysis Corp.). An H&E slide was evaluated for the tumour area by a pathologist and infiltrating tumour was circled. An unstained section was then pretreated and probed using standard treatment procedures. Gene amplification was assessed by analysing a minimum of 20 non-overlapping nuclei, containing at least one orange and one green signal. The ratio of orange to green signals was calculated. A ratio of ⩾2.0 is considered as amplified.

For the HER3 analysis, tumour areas of interest were identified and microdissected from the paraffin-embedded tissue section. The collected cells were digested and total RNA was purified from the sample using Qiagen RNeasy FFPE kit (Qiagen, Germantown, MD, USA). Total RNA was converted to cDNA using SuperScript III from Invitrogen (Carlsbad, CA, USA). Sample and control cDNA were subjected to real-time RT–PCR using an Applied Biosystems 7900HT (Applied Biosystems, Foster City, CA, USA). The expression values were calculated as a ratio of the target to reference gene transcript.

## Results

### Patient characteristics

A total of 38 patients were enrolled between 21 December 2007 and 6 November 2009 from 12 participating sites. Table 1 lists the demographic and clinical characteristics of the patients. Per protocol, 5 of the 38 patients were not evaluable for best response; 2 withdrew consent after receiving one cycle and 3 additional patients had treatment toxicity and were changed to other therapies. Most patients were men (87%) and the median age was 59. All patients had an ECOG PS of 0 or 1 (PS 0=60%, PS1=40%). In all, 95% of patients had metastatic disease and 5% were considered unresectable by a qualified thoracic surgeon. In total, 32% of patients had distal oesophageal cancers (Siewert class I) and 68% had tumours from the GEJ and cardia (Siewert class II and III). Altogether, 13% of patients had a history of an oesophagectomy, 16% had received previous radiation and 21% had received previous chemotherapy (either neoadjuvant or adjuvant). Of the eight patients that previously received chemotherapy, two received adjuvant chemoradiation (5-FU based), four received neoadjuvant chemoradiation (5-FU based) and two received neoadjuvant chemo alone (ECF).

### Treatment efficacy

Of the 33 patients who were evaluable for response per protocol, there were 2 CRs and 15 PRs for an ORR of 51.5% (95% CI, 34.5–68.6%). In all, 15 responses have been formally confirmed resulting in a confirmed ORR of 45%. When the five patients who did not undergo repeat evaluation are considered as progressive disease, the ORR is 45% (95% CI, 30.0–61%). An additional 11 patients (29%) achieved stabilisation of their disease. The median follow-up for patients on study was 14.1 months.

**Figure 1:**
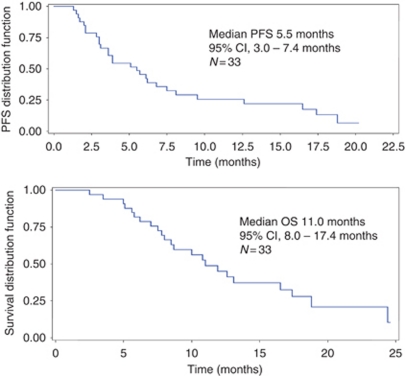
Progression-free survival (PFS) and overall survival (OS) (Kaplan–Meier estimates).

Figure 1 demonstrates the Kaplan–Meier estimates of PFS and OS. Median PFS was 5.5 months (95% CI, 3.1–7.5 months) and median OS was 11.0 months (95% CI, 8.0–17.4 months).

### Safety data

Adverse events associated with FOLFOX/erlotinib therapy are listed in Table 2. In all, 84% of the AEs were grade 1–2 with 16% being grade 3–4. The most common grade 3–4 toxicities seen were non-haematologic: diarrhoea (24%), anorexia (13%), nausea/vomiting (11%), skin rash (8%), fatigue (11 %) and peripheral neuropathy (8%). Haematologic grade 3–4 toxicities consisted of 13% of patients experienced grade 3 neutropenia with one patient (3%) having febrile neutropenia.

Erlotinib dose was modified in 39% of patients and were usually dose reductions to 100 mg.

### Biomarker analysis

Tumour biopsies were available in 36 out of 38 (95%) patients enrolled. Samples were analysed for mutations in EGFR, KRAS, BRAF and PI3 kinase. In addition, amplification by FISH was performed for EGFR and HER2 and expression by RT–PCR for HER3 levels. Table 3 lists the frequency of the molecular alterations. The frequency of mutations in KRAS was 8%, and only one patient had a mutation in PI3 kinase. The EGFR and BRAF mutations were not detected. The rates of EGFR and HER2 amplification were 8% and 19%, respectively. Given the small sample size, there was no predictive value to either RR, PFS or OS with any of the biomarkers analysed.

## Discussion

Historically, clinical trials in upper GI tract malignancies have been lumped together both histologically (squamous cell and adenocarcinomas) or location (oesophagus, GEJ and distal stomach). Over the last several years, it has become clear that these represent different biological entities both in terms of epidemiology, molecular biology and trends of incidence. In Western Europe and North America, the rates of GEJ cancers have increased, whereas those gastric tumours, which are H Pylori associated have fallen. Few trials have been reported specifically in patients with GEJ cancers (Stahl *et al*, 2009). To our knowledge, the trial reported herein is the first to examine the addition of a targeted agent to chemotherapy in this specific subset of upper GI cancers.

This phase II trial examined the efficacy and safety of the combination of FOLFOX chemotherapy and erlotinib in patients with advanced adenocarcinoma of the oesophagus and GEJ. The primary endpoint in this trial, objective RR was 51%, which was similar to those recently reported in combination clinical trials with chemotherapies and anti-EGFR monoclonal antibodies in similar patient populations (Han *et al*, 2009; Pinto *et al*, 2009; Lordick *et al*, 2010). However, these trials contained different chemotherapy backbones and as recently reported in oesophageal adenocarcinoma, the monoclonal antibody cetuximab may be less biologically active in this group that anti-EGFR tyrosine kinase inhibitors (Gold *et al*, 2010). Interestingly, in a recently reported randomised phase II clinical trial evaluating various chemotherapy regimens with cetuximab, the highest PFS and OS were associated with the FOLFOX combination (Enzinger *et al*, 2010). Furthermore, our secondary endpoints of PFS and OS of 5.5 and 11.0 months, respectively, are also consistent with recent reports.

The combination of FOLFOX and erlotinib was generally well tolerated with few grade 3–4 toxicities consistent with a previous phase I study that examined this combination (Hanauske *et al*, 2007). Not surprisingly, an acneiform rash was the most common toxicity attributed to the addition of erlotinib in this trial. The protocol recommended aggressive treatment of the rash, which may explain the low rate (8%) of grade 3 toxicities. The most common grade 3 or 4 toxicities seen were diarrhoea and dehydration (24%) but these toxicities rarely required hospitalisation and was significantly reduced when erlotinib was decreased to 100 mg. However, based on these findings, a starting dose of erlotinib at 100 mg could be considered in further studies. In addition, 8% of patients experienced grade 3 oxaliplatin-related peripheral neuropathy. While 39% of patients had a dose reduction to 100 mg of erlotinib, there were no patients who stopped erlotinib for greater than a 2-week period because of toxicity.

This study sought to examine some biological surrogates of EGFR pathway activation including EGFR and KRAS mutation status, EGFR and HER2 amplification and HER3 overexpression. Previous reports in lung cancer patients have demonstrated that clinical benefit from tyrosine kinase inhibitors is greater in patients whose tumours harbour an EGFR mutation (Lynch *et al*, 2004; Tsao *et al*, 2005; Maemondo *et al*, 2010). In our analysis, we did not detect any mutations in exons 18, 19 and 21. Although mutations in EGFR have been reported in oesophageal cancers, in one of the largest analyses to date, Dragovich *et al* (2006) did not detect any EGFR mutations in GEJ cancers (Guo *et al*, 2006; Kwak *et al*, 2006; Altiok *et al*, 2010). While we did detect evidence of EGFR gene amplification in 8% of patients (3 out of 36), consistent with previous reports, the small numbers precluded any correlation with clinical response (Janmaat *et al*, 2006).

On the basis of the recently reported results of the role of the HER2 oncogene in gastric and oesophageal cancers, we sought to investigate the frequency and correlation of HER2 amplification. Our results indicate that 19% (7 out of 36) had evidence of HER2 amplification by FISH. The frequency of this alteration is consistent with the large randomised clinical trial of trastuzumab to chemotherapy (ToGA) trial (Bang *et al*, 2010). Not surprisingly and consistent with previous reports in other cancers, there was no correlation in patients treated with an EGFR inhibitor in our trial.

Recent reports have also implicated HER3 as having a critical role as a co-receptor for HER2 amplification as well as a marker for sensitivity to EGFR inhibition (Alimandi *et al*, 1995; Erjala *et al*, 2006; Engelman *et al*, 2007). Several studies have examined the rates of HER3 expression in gastric cancer by immunohistochemistry with one study showing correlation with a worse prognosis (Hayashi *et al*, 2008; Zhang *et al*, 2009). More recent studies have used RT–PCR to assess the rates of HER3 expression and using this technique, we found that 4 out of 36 tumours overexpressed HER3 (Witta *et al*, 2009). However, there was neither a correlation of HER3 with response nor an association with HER2 amplification by FISH. The role and most appropriate assays to measure HER3 clearly require further investigation.

Mutations in KRAS have recently become established as a negative predictive marker for the benefit of EGFR monoclonal antibodies in advanced colorectal cancer (Amado *et al*, 2008; Van Cutsem *et al*, 2009). Tajima *et al* (2007) compared the incidence of KRAS mutations in gastric cardia adenocarcinomas and distal stomach cancers and reported a 7% positive mutation rate in both groups. Although few trials have looked specifically at GEJ tumours, the 8% rate of KRAS mutation in this trial is consistent with reports in oesophageal and gastric cancers (Arber *et al*, 2000; Lord *et al*, 2000). In this trial, all three patients with a mutation in KRAS did achieve an objective PR but the small sample size precluded any statistical correlation.

This study had several limitations with the major weakness being that it was a single-arm, non-comparative study. Unfortunately, the small numbers of patients and the diversity of these diseases have made any biomarker correlations a great challenge. However, our study was unique in those specifically excluded patients with distal gastric cancer, a subset of this disease that has distinct epidemiology, molecular alterations and prognosis. Our study also adds to those that have explored translational correlatives in this rare group of cancers (Janmaat *et al*, 2006; Rojo *et al*, 2006; Han *et al*, 2009). With the emergence of GEJ cancer as an increasingly recognised distinct entity, it is anticipated that more clinical trials will focus on the subsets of this malignancies. This study treated only adenocarcinoma of the oesophagus and GEJ, a subgroup that based on previous studies may be more sensitive to EGFR inhibition. Ongoing studies that are currently randomising patients to chemotherapy with or without EGFR inhibitors will clarify the true biologic activity of these agents.

## Figures and Tables

**Table 1:** Patient demographics and clinical characteristics

**Demographic or characteristic**	**Patients (*N*=38)**
Median age, years (range)	59 (36–79)
Female: male, number	5:33
ECOG PS: 0:1	23:15
Oesophagus: GEJ	12:26
Unresectable: metastatic	2:36
	
Site of metastasis	
Abdominal nodes: liver: lung: bone: other	28:19:12:5:4
	
Previous treatment:	
Surgery: yes: no	5:33
Neoadjuvant or adjuvant chemo: yes: no	8:30
	
Previous chemo regimens:	
Fluoropyrimidines/platinum (with adjuvant radiation)	2
Cisplatin/5-FU based (with neoadjuvant radiation)	4
Epirubicin/cisplatin/5-FU	2
Previous radiation therapy: yes: no	6:32
Neoadjuvant:adjuvant	4:2

Abbreviations: ECOG PS=Eastern Cooperative Group performance status; 5-Fu=5-fluorouracil; GEJ=gastro-oesophageal junction.

**Table 2:** Common grade 3 and 4 toxicities of the combination of FOLFOX and erlotinib (*N*=38)

**AE**	**Grade 3 (%)**	**Grade 4 (%)**
*Non-haematologica*		
Diarrhoea/dehydration	9 (24)	
Anorexia	5 (13)	
Nausea/vomiting	3 (8)	1 (3)
Rash	3 (8)	
Fatigue	3 (8)	1 (3)
Peripheral neuropathy	3 (8)	
		
*Haematologica*		
Neutropenia	5 (13)	
Neutropenic fever		1 (3)
		
*Lab only*		
Hypokalaemia	2 (5)	
AST/ALT elevation	2 (5)	

Abbreviations: AE= adverse event; ALT=alanine aminotransferase; AST=aspartate aminotransferase.

**Table 3:** Overall rates of KRAS, EGFR, BRAF, PI3Kinase mutations, EGFR and HER2 amplification by FISH and HER3 overexpression

**Biomarker**	**Yes *N* (%)**	**No *N* (%)**
KRAS mutation	3 (8)	33 (92)
EGFR mutation	0 (0)	36 (100)
BRAF mutation	0 (0)	36 (100)
PI3Kinase mutation	1 (3)	35 (97)
EGFR amplified (FISH)	3 (8)	33 (92)
HER2 amplified (FISH)	7 (19)	29 (81)
HER3 overexpressed (RT–PCR)	4 (11)	32 (89)

Abbreviations: BRAF=v-raf murine sarcoma viral oncogene; EGFR=epidermal growth factor receptor; FISH=fluorescence *in situ* hybridisation; HER2=human epidermal growth factor receptor 2; KRAS=Kirsten rat sarcoma viral oncogene; PI3Kinase=phosphatidylinositol 3 kinase; RT–PCR=reverse transcriptase-PCR.
